# 

**Published:** 1895-08

**Authors:** 


### CONTENTS—AUGUST, 1895.—VOL. XI., NO. 2.

ORIGINAL COMMUNICATIONS—

Clinical Microscopy for the General Practitioner. Hugh H. Young, A. M., M. D., San Antonio . . . . . 53
Gangrene. D. Dupre, M. D., Oak Cliff . . . . . 60
A Modern Treatment of Hemorrhoids. T. J. Bennett, M. D., Austin 64
How to Cure Puerperal Eclampsia. Emory Lanphear, M. D., Ph. D., St. Louis, Mo . . . . . 70

CURRENT MEDICAL LITERATURE—

Department of Practice of Medicine. Edited by Prof. Allen J. Smith, Galveston . . . . . 73

CORRESPONDENCE—

Dr. Peoples' Miasmatic Paralytic Fever. Dr. Thomas A. Pope . . . 75
Dr. Karnes' Case. Dr. W. W. Walker . . . . . 76

SOCIETY NOTES—

Medico-Legal Congress . . . . . 77
Colorado State Medical Association . . . . . 79

ABSTRACTS AND SELECTIONS—

Medical Liberalism. . . . . . 79

EDITORIAL—

Dr. Merriman on Mandrakes . . . . . 81
The "Decline and Fall Off" . . . . . 84
Death of Dr. Josephus Cummings . . . . . 86

MEDICAL NEWS AND MISCELLANY—

Numerous Paragraphs of Interest . . . . . 87

BOOK NOTICES— . . . . . 95
PUBLISHERS' NOTES . . . . . 97

